# Evolution of ontogenic change in color defenses of swallowtail butterflies

**DOI:** 10.1002/ece3.4426

**Published:** 2018-09-03

**Authors:** Nikhil Gaitonde, Jahnavi Joshi, Krushnamegh Kunte

**Affiliations:** ^1^ National Center for Biological Sciences Tata Institute of Fundamental Research Bengaluru India; ^2^ Manipal Academy of Higher Education (MAHE) Manipal India

**Keywords:** animal communication, coevolution, color defense, life history, predation, signal environment

## Abstract

Natural selection by visually hunting predators has led to the evolution of color defense strategies such as masquerade, crypsis, and aposematism that reduce the risk of predation in prey species. These color defenses are not mutually exclusive, and switches between strategies with ontogenic development are widespread across taxa. However, the evolutionary dynamics of ontogenic color change are poorly understood. Using comparative phylogenetics, we studied the evolution of color defenses in the complex life cycles of swallowtail butterflies (family Papilionidae). We also tested the relative importance of life history traits, chemical and visual backgrounds, and ancestry on the evolution of protective coloration. We found that vulnerable early‐ and late‐instar caterpillars of species that feed on sparsely vegetated, toxic plants were aposematic, whereas species that feed on densely vegetated, nontoxic plants had masquerading and cryptic caterpillars. Masquerading caterpillars resembled bird droppings at early instars and transitioned to crypsis with an increase in body size at late instars. The immobile pupae—safe from motion‐detecting, visually hunting predators—retained the ancestral cryptic coloration in all lineages, irrespective of the toxic nature of the host plant. Thus, color defense strategy (masquerade, crypsis, or aposematism) at a particular lifestage in the life cycle of swallowtail butterflies was determined by the interaction between life history traits such as body size and motion levels, phytochemical and visual backgrounds, and ancestry. We show that ontogenic color change in swallowtail butterflies is an adaptive response to age‐dependent vulnerability to predation.

## 1 INTRODUCTION

Natural selection imposed by predation has led to the evolution of antipredatory, morphological, behavioral, and life historical defenses in prey species (Abrams, 2000; Brodie & Brodie, 1999; Langerhans, 2007; Schmidt, 1990). Prey species may also reduce the risk of predation from visually hunting predators using color defense strategies such as masquerade, crypsis, and aposematism (Booth, 1990; Caro, Sherratt, & Stevens, 2016; Cuthill et al., 2017; Higginson & Ruxton, 2010; Lichter‐Marck, Wylde, Aaron, Oliver, & Singer, 2014; Skelhorn, Rowland, Speed, & Ruxton, 2010; Speed, 2000). Masquerading prey resemble some inedible objects (at times, objects aversive to their predators, such as bird droppings) in their natural environment and are misidentified by predators, whereas cryptic prey avoid detection by matching the color and pattern of their body with those of the background (Duarte, Flores, & Stevens, 2017). Aposematic species, on the other hand, are usually chemically defended and therefore are distasteful and unprofitable and advertise their unpalatability through warning coloration to predators that learn to avoid harmful prey (Mappes, Marples, & Endler, 2005; Mukherjee & Heithaus, 2013; Stevens & Ruxton, 2012). Aposematic species are under selection to increase their conspicuousness and stand out from the background as warning coloration works effectively when apparent, helping predators to detect and avoid it precisely and rapidly (Caro et al., 2016; Finkbeiner, Briscoe, & Reed, 2014; Mappes et al., 2005; Speed, 2000; Speed, Brockhurst, & Ruxton, 2010; Stevens & Ruxton, 2012). Color defense strategies are not mutually exclusive, and organisms may employ different strategies across ecological contexts or at different lifestages that experience differential predation risk (Booth, 1990; Caro et al., 2016; Grant, 2007; Nyboer, Gray, & Chapman, 2014; Valkonen et al., 2014; Wilson, Heinsohn, & Endler, 2007). Change in coloration corresponding to ontogenic shifts is known as ontogenic color change, which is widespread across taxa (Booth, 1990; Caro et al., 2016; Grant, 2007; Nyboer et al., 2014; Wilson et al., 2007). However, its evolutionary dynamics are poorly understood (Caro et al., 2016; Cuthill et al., 2017).

Ontogenic color change seems to be a life historical response to changing levels of natural selection on distinct lifestages of complex life cycles, where vulnerability to predation varies across ontogeny (Abrams, 2000; Bond & Kamil, 2002; Caro et al., 2016; Higginson & Ruxton, 2010; Merilaita, 2003; Gadgil & Bossert, 1970). For example, predation risk differs across lifestages due to progressive increase in body size with development, wherein a small body size is difficult to detect than a large one. Similarly, predation risk also varies with the level of movement associated with a lifestage as many visually hunting predators (reptiles, birds, and small mammals) detect prey using motion. In addition to size and motion levels which often interact to determine predation pressure on a lifestage, habitat shifts, activity pattern, and diversity of predators are important factors which influence age‐dependent vulnerability (Caro et al., 2016; Johansen, Tullberg, & Gambrale‐Stille, 2011). Color defenses that reduce predation risk are therefore expected to change with changes in predation risk giving rise to ontogenic color change (Caro et al., 2016). For example, the caterpillars of *Saucrobotys futilalis* or *Acronicta alni* switch from being cryptic at early instars, to aposematic at late instars. The switch in their coloration occurs with the accumulation of toxins from their diet, and they increasingly turn conspicuous with progressive lifestages to become aposematic. The switch in the color defense seems to be driven by increased vulnerability of late‐instar caterpillars due to their large body size, high activity, and increased movement before pupation (Caro et al., 2016; Grant, 2007; Johansen et al., 2011). Although individual factors such as body size and motion, differential predation risk, and diet, which predict animal coloration, are identified and well studied, the interaction between factors and their influence on the evolution of animal coloration is missing.

An additional and important factor that influences vulnerability to predators is the visual background (Caro et al., 2016; Duarte et al., 2017), whose role as selective agent on protective coloration has been widely demonstrated (Bond & Kamil, 2002; Higginson & Ruxton, 2010; Merilaita, 2003; Prudic, Oliver, & Sperling, 2007). Several examples, such as fluctuations in morph frequencies with change in visual backgrounds, higher survival and recapture of morphs against matching than nonmatching backgrounds, and the specific concealment of prey to the vision of their predators, demonstrate the survival benefit of background matching (Chiao, Wickiser, Allen, Genter, & Hanlon, 2011; Duarte et al., 2017; Hultgren & Mittelstaedt, 2015; Stuart‐Fox, Moussalli, & Whiting, 2008). For color‐changing animals, visual backgrounds and chromatic contrast are important as they can be used to either enhance detection in case of aposematic species (by increasing contrast and standing out from the background color), or prevent recognition by masquerading some inedible object (caterpillars masquerading as bird dropping are conspicuous against vegetation and are easily detected, but not recognized as prey), or prevent detection by decreasing the contrast of the body color and blend into the background color in case of cryptic prey. Although evolution of aposematic warning coloration is well known, the context under which prey opt for either masquerade or crypsis to conceal themselves is poorly understood. For instance, why do caterpillars masquerading as bird droppings switch to become cryptic or aposematic at late instars? Increase in body size and motion with development give away their cover and severely constrain protection by masquerade, but a comparative analysis of these factors and their relative strengths is unknown. The distinct, free‐ranging lifestages of swallowtail butterflies offer an opportunity to test the interaction of lifestage‐associated traits such as body size and motion along with their visual backgrounds to better understand how color patterns and body forms are adaptively selected (Cuthill et al., 2017; Duarte et al., 2017; Wilbur, 1980).

The globally distributed swallowtail butterflies (family Papilionidae) serve as an excellent model to study the evolution of defensive coloration. Their distinct lifestages in a complex life cycle experience differential predation risk from visually hunting predators such as insectivorous birds, reptiles, and mammals that detect prey using body size and motion (Greeney, Dyer, & Smilanich, 2012; Johansen et al., 2011; Lichter‐Marck et al., 2014). Swallowtail caterpillars progressively increase in body size with development and in motion levels, due to increase in foraging demands and during pupation, which attracts attention of motion‐detecting, visually hunting predators. On the other hand, the immobile pupal stage is relatively safe due to the absence of motion (Caro et al., 2016; Greeney et al., 2012; Speed et al., 2010; Wiklund & Sillén‐Tullberg, 1985). The contrasting lifestage vulnerability of caterpillars and pupae provides an ideal opportunity to test changes in age‐dependent vulnerability as a driver for ontogenic color change, as well as understand the adaptive links between form and color. Apart from the differential predation risk on complex life cycles of Papilionidae, their strong and specific interactions with host plants provide an ideal setup to concurrently test the influence of visual backgrounds on color defense strategies (Condamine, Sperling, Wahlberg, Rasplus, & Kergoat, 2012; Dyer et al., 2007; Ehrlich & Raven, 1964; Nishida, 2002; Prudic et al., 2007; Scriber, Tsubaki, & Lederhouse, 1995; Thompson, 1998) The host plants of Papilionidae play a crucial role in the evolution of color defenses by determining two important aspects of vision‐based predation: (a) They provide caterpillars with toxins, which swallowtails are unable to synthesize by themselves and become unprofitable (Nishida, 2002), and (b) they determine the apparency of lifestages to predators by defining background properties such as ambient light, dominant color, and chromatic contrast by virtue of their gross morphology, which influences the efficacy of all color defenses (Booth, 1990; Caro et al., 2016; Prudic et al., 2007; Tullberg, Merilaita, & Wiklund, 2005). Papilionidae use nine plant families as their larval hosts, viz. (a) Aristolochiaceae, (b) Apiaceae, (c) Crassulaceae + Saxifragaceae (sometimes considered a single family), (d) Papaveraceae, (e) Zygophyllaceae, (f) Rutaceae, (g) Lauraceae, (h) Annonaceae, and (i) Fabaceae (Condamine et al., 2012; Dyer et al., 2007; Fordyce, 2010; Gandon, Ebert, Olibieri, & Michalakis, 1998; Thompson, 1998). The first five host plant families are either vines, succulents, and herbs and are sparsely vegetated (Condamine et al., 2012; Ehrlich & Raven, 1964; Prudic et al., 2007). The remaining four host plant families are large woody trees with dense foliage and are nontoxic, and therefore, all the lifestages (caterpillars, pupa, and adults) of species associated with a nontoxic host plant are palatable to the vertebrate predators of Papilionidae (swallowtail butterflies cannot synthesize toxins de novo*,* but some species groups sequester toxins from larval host plants) (Condamine et al., 2012; Ehrlich & Raven, 1964; Prudic et al., 2007). Thus, host plants of Papilionidae provide contrasting visual and phytochemical backgrounds for the evolution and maintenance of diverse color defense strategies. In the genus *Papilio*, the signal environment (visual background) better predicted the evolution of aposematic coloration than diet or chemical specialization of lineages, highlighting visual background as a selective agent for warning coloration (Prudic et al., 2007). We test whether this prediction holds true at a larger phylogenetic scale and attempt to understand the roles that diet and visual backgrounds generally play in the evolution of protective coloration. This perspective is important as it gives a holistic understanding of color defenses and a comparative account of factors that influence the evolutionary trajectory of all major types of color defenses.

In this paper, we first quantitatively assess the natural history‐based classification of color defense strategies of Papilionidae and then examine the role of ecological and evolutionary factors such as life history, signal environment, diet and phylogenetic constraints on the evolution of color defenses, and their ontogenic shifts, in the life cycles of swallowtail butterflies.

## 2 METHODS

### 2.1 Data collection

**Figure 1:**
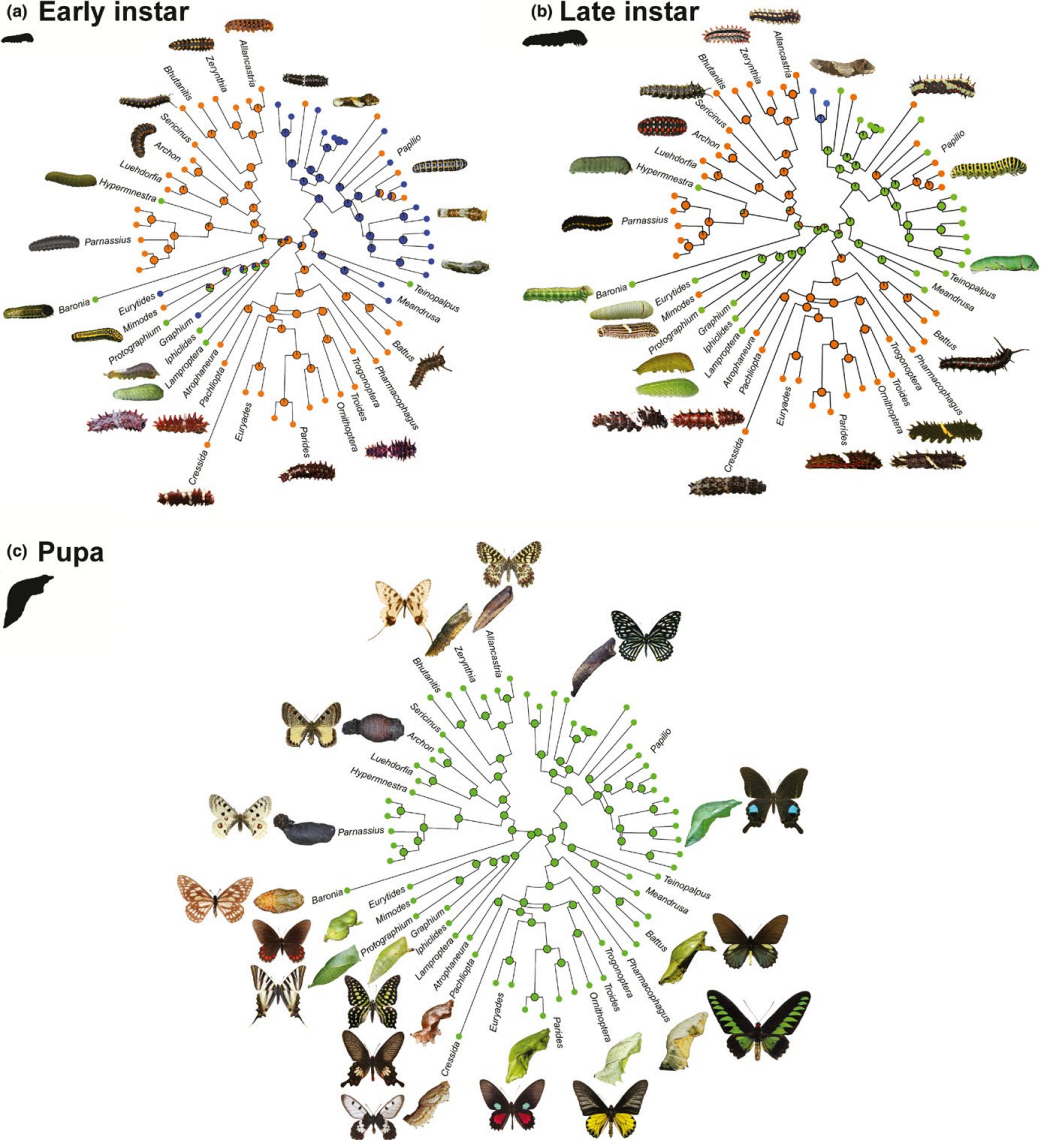
Character mapping of color defenses at various lifestages on the global molecular phylogeny of swallowtail butterflies (Papilionidae). Lineages with masquerading caterpillars are depicted in blue (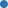), cryptic in green (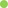), and aposematic in orange (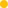). Pie charts at internal nodes show proportional Bayesian probability of color defense strategies. Early (a) and late (b) instars are depicted by the size of the caterpillar silhouettes and are motile, whereas pupae (c) are immobile. Images of caterpillars, pupae, and butterflies used to depict color defense phenotypes were taken from Wikimedia Commons and other Web resources (Appendix, used with permission where required). (a) Vulnerable early‐instar caterpillars were predominantly masquerading or aposematic with only two lineages exhibiting crypsis. (b) Lineages masquerading at early instars switched to crypsis or aposematism with increase in body size at late instars. (c) All lineages retained their ancestral crypsis at the immobile and therefore less vulnerable pupal stage

We examined defense strategies of Papilionidae (all 22 genera within Papilionidae with 63 representatives of all major lineages or species groups) at three lifestages: early instar, late instar, and pupa (Figure 1). We excluded adult defensive coloration because aposematism and mimicry are particularly well studied in adult swallowtails (Kunte, 2008, 2009; Scriber et al., 1995). We collated images of caterpillars and pupae from peer‐reviewed, online resources and from the published literature (Appendix). The defense strategy (phenotype) at each lifestage was discretely scored as either masquerading, cryptic, or aposematic. We used representatives of each genus for trait mapping as defensive strategies are usually uniform within genera and species groups, although some exceptions exist, as our analysis will also reveal (Figure 1). We assessed the natural history‐based classification of color defense strategies by quantifying the colors of preadult lifestages and their respective backgrounds.

We collected information on larval host plant families, their growth type, and presence of toxic phytochemicals from multiple sources such as the angiosperm phylogeny group (http://www.mobot.org) and published research articles for all genera within Papilionidae. We scored host plant characteristics discretely, as either toxic or nontoxic, and as either sparsely (vines, succulents, herbs, and shrubs) or densely vegetated (woody trees) following previous studies (Condamine et al., 2012; Prudic et al., 2007). The discrete binning of Papilionidae host plants as toxic and nontoxic, and sparsely and densely vegetated, is based on several strong morphotaxonomic characters of plant families. Among the host plant families of Papilionidae, Aristolochiaceae, Crassulaceae + Saxifragaceae (sometimes considered a single family), Papaveraceae, and Zygophyllaceae are confirmed to contain toxins, most probably to protect their sparse vegetation during their short life spans against herbivory. On the other hand, woody tree families such as Apiaceae, Rutaceae, Lauraceae, Annonaceae, and Fabaceae do not possess toxins (chemicals toxic to vertebrate predators of Papilionidae such as birds and small mammals) but have morphological defenses such as thick leaves, thorns, and various induced defenses that reduce herbivory through their long life spans. As in most antagonistic interactions, the toxicity of the host plant is determined by the cost–benefit ratio of possessing a chemical defense. Therefore, plants with sparse vegetation and short life spans are observed to have strong antiherbivory chemical defenses, whereas the long‐lived densely vegetated tall woody trees need not possess chemicals toxic to the vertebrate predators of Papilionidae possibly because many omnivores are seed dispersers of these plant families. The coevolutionary interaction between woody plant families and their vertebrate seed dispersers presumably constrains plants from evolving toxins that may harm their vertebrate partners. Therefore, caterpillars feeding on woody plants are devoid of chemicals harmful to their vertebrate visually hunting predators, in whose response color defenses have evolved. With regard to morphology, the growth type of the plant family is conserved within a family. Host plant families such as Aristolochiaceae, Crassulaceae + Saxifragaceae (sometimes considered a single family), Papaveraceae, and Zygophyllaceae grow as vines, succulents, and herbs and have stunted growth with simple leaves often alternately arranged, giving rise to sparse foliage, whereas woody plant families are tall and profusely branched and have compound leaves often arranged in opposition, creating a dense foliage. The difference in the gross morphology of host plant families of Papilionidae as sparsely and densely vegetated is responsible to provide distinct visual backgrounds to the caterpillars feeding on them. Sparse vegetation allows more light, and caterpillars feeding on them are exposed compared to ones feeding on dense vegetation where shade and overlapping foliage provide refuge from predators. We found that the character scheme based on the leaf size, growth type, and gross morphology of host plant families of Papilionidae prepared by Prudich et al. is appropriate to investigate broad‐scale evolutionary patterns, and we adopt the same in our analyses. Thus, the host plants of Papilionidae provide contrasting phytochemical and visual backgrounds for the evolution of color defenses of Papilionidae against their vertebrate visually hunting predators.

Papilionidae have strict oviposition and larval food choice, and once the female lays eggs on plants belonging to the respective host plant family, the caterpillars usually feed on the same plant until pupation. While most caterpillars pupate on or near their host plant, some temperate lineages move away from the plants and pupate on the ground. The diversification of Papilionidae into varied habitats and on different host plant families acts as a natural experiment where some Papilionidae lineages feed on toxic and sparsely vegetated plants, whereas the rest are under nontoxic, densely vegetated backgrounds, and some lifestages (early‐ and late‐instar caterpillars) are motile, whereas pupae are nonmotile, therefore providing an ideal opportunity to test the interaction between phytochemicals, visual backgrounds, and age‐dependent vulnerability on the evolution of color defenses.

### 2.2 Quantification of color defenses of Papilionidae

Rigorous characterization of natural coloration and defense strategies of butterfly caterpillars and pupae has not been attempted because of inherent limitations of using certain kinds of scientific methods such as spectroscopy and controlled predation experiments on a large number of species distributed across the world. However, there is considerable amount of natural history information available on early stages and larval host plants of swallowtail butterflies. Majority of the caterpillars and pupae of Papilionidae have been documented with photographs (although under variable field conditions and camera equipment) and made available in the scientific literature and scholarly Web sites (Appendix). We compiled this information and scored color defense phenotypes of swallowtails in the following manner: (a) The color defense strategy of the species was discreetly scored as masquerade, crypsis, or aposematism based on field guides, online resources on butterfly life cycles, natural history reports, and research articles (Appendix). (b) We downloaded good‐quality (minimal shadows and no burn) images of lifestages captured at close range (full‐frame images, captured approximately within 5 m) and under natural conditions. We separated the background by selecting along the outline of the lifestage and saved the images as portable network graphics (.png) files. (c) We then extracted the color gamut of the sample image using an online TinEye© color extraction application. The application gave a similarity rank, a weight factor, a color name, and a color class for each color and returned the relative proportion and RGB values of all colors constituting the image and is detailed here at https://services.tineye.com/developers/multicolorengine/methods/extract_image_colors.html. (d) We identified the primary color of the lifestage and background (relative proportion >25%) and plotted their red, green, and blue values, in RGB color space (255,255,255) (see Supporting Information Figures [Link], [Link], [Link]). (e) We then calculated the color difference of a lifestage by subtracting the RGB value of primary color of the lifestage from that of its background as “*delta RGB*” (|*delta RGB| *= dominant color of background in RGB – dominant color lifestage in RGB) and performed a principal component analysis on the delta RGB values, grouping color defenses as either apparent (masquerade and aposematism) or nonapparent (crypsis). Note that the delta RGB is not a color, but an estimate of the visual contrast between the primary color of the caterpillar and its background (a triplet of delta values of R, G, and B). To distinguish between apparent (aposematism and masquerade) and nonapparent (crypsis) color defense strategies at both early‐ and late‐instar caterpillars, we compared the PC1 score (PC1 scores explained >80% of variance at both early‐ and late‐instar caterpillar stages) of species with apparent and nonapparent color defenses using Mann–Whitney *U*‐test. As all pupae are considered to be cryptic, we validated whether they matched their backgrounds by comparing their R, G, and B values of the pupa with those of the background using Kruskal–Wallis test, followed by appropriate post hoc tests.

We selected images captured only under natural conditions, and all the images used in the analysis are linked to their source in Appendix. Although the images were taken using different cameras and lighting conditions, the potential errors in color measurement were minimized by subtracting the RGB value of the dominant color of the lifestage from its respective background. *Delta RGB* is a fair estimate of the visual color contrast and was further used to validate the natural history‐based classification of color defenses. Based on the color theory, we expected aposematic and masquerading (as bird droppings) caterpillars to be apparent from the background (high *delta RGB* value), whereas cryptic lifestages to be camouflaged have low *delta RGB* value. We effectively captured major differences in the color defense phenotypes, and if any minor differences were masked, they might not affect the overall evolutionary patterns in a significant manner as the comparisons we make are at the level of distinct lineages and genera. Distinct Papilionidae lineages have little variation in the color defense phenotypes at the level of species, as the color defenses are influenced by the tight coevolutionary interaction between swallowtail clades and host plant families which determine the type of color defense in swallowtail caterpillars. It is rather impossible to take more precise measurements of three distinct lifestages of a globally distributed hyperdiverse group.

### 2.3 Phylogeny reconstruction and character evolution

We rebuilt the global swallowtail phylogeny from published sequences as the published phylogenetic tree was not available on any data repository long after the papers were published (Condamine, Sperling, & Kergoat, 2013; Condamine et al., 2012). Our phylogeny contained identical taxa (Condamine et al., 2012, 2013) and the same three mitochondrial (*cytochrome oxidase I*,* tRNA leucine*,* cytochrome oxidase II*) and one nuclear (*elongation factor 1*‐α) gene sequences (2.3 kilobase) for 164 taxa representing all the known Papilionidae genera. We downloaded published DNA sequences from NCBI and assembled those using Geneious 7. We used a Bayesian approach to reconstruct phylogeny with MrBayes 3.2 on partitioned data (mtDNA and nuclear) with GTR+I+γ model of sequence evolution. We ran the program for 50 million generations wherein sampling was made for every 1,000 generations. We used split frequency below 0.01 as a measure to assess stationarity and to set the burn‐in. We built a consensus tree using the remaining trees. The tree was rooted using out‐groups as per the previously published phylogeny (out‐groups: *Colias, Vanessa, Coenonympha, Libythea,* and *Pyrgus*). We found that there was little variation below the level of genera/species groups in the color defense phenotypes of preadult lifestages. We therefore chose a representative for each distinct lineage/genera/species group (63 taxa representing all distinct lineages and genera) for our trait mapping study and dropped the rest of the tips.

Ancestral character mapping of color defenses at each lifestage was performed in *R* using the function “simmap,” package *phytools* (Revell, 2012). We simulated character mapping 2,000 times as the mean probability of a state at a particular node for the most phenotypically variable lifestage stabilized around the 2,000th run (Supporting Information Figure S4). We ran a phylogenetic generalized linear model to test the influence of different factors such as visual and phytochemical backgrounds and temperate or tropical distribution of species, on the evolution of color defenses at late‐instar caterpillars using the function “phyloglm,” package *phyloglm* (Ho and Ané, 2014; Ho et al., 2016). As all phylogenetic regression methods for discrete data can only analyze binary data, we grouped color defense phenotypes as apparent (aposematic or masquerading) and nonapparent (cryptic). As phytochemical and visual backgrounds were correlated (*R* ^2^ = 0.9, *p *< 0.01, phylogenetic regression, *z* = 4.27, *p *<* *0.001), we used only one of them (visual background) as a predictor (phyloglm: phenotype ~ visual background + distribution, method = logistic_MPLE) (Ho et al., 2016).

## 3 RESULTS

### 3.1 Quantification of color defenses of Papilionidae

Our sole objective of quantification of color defenses was to validate the natural history‐based classification of species as masquerading, aposematic, or cryptic using color as an independent measure. We expected the apparent color defenses of aposematism and masquerade to cluster together as they are conspicuous and stand out from the background, whereas cryptic species blend into the background. We calculated *delta RGB* as a measure of conspicuousness and used it to distinguish between strategies. At the early‐ and late‐instar caterpillar stages, apparent and nonapparent caterpillars were significantly different along the PC1 color axis (early instar: *W* = 205, *p *=* *0.002; late instar: *W* = 627, *p *<* *0.001). At the pupal stage, no significant difference was observed between the dominant RGB values of lifestage and their respective backgrounds (*p *>* *0.05 for R, G, and B channels) indicating camouflage in pupae by matching the color of the background.

Species that were apparent but showcased only shades of white or gray were considered to masquerade, especially as they also matched the color palette of an image of a bird dropping in RGB colorspace (Supporting Information Figure S1). The rest were considered aposematic as they had conspicuous secondary colors such as red and yellow that are associated with warning coloration (Stevens & Ruxton, 2012; Supporting Information Figure S1). The natural history‐based classification of color defense strategies was corroborated by statistically analyzing the relative colors against their background giving confidence in assigning character states to species as masquerading, cryptic, and aposematic which are further used in phylogenetic analysis (Figure 1) to understand the evolution of color defense strategies.

### 3.2 The evolution of color defense strategies and ontogenic color change

**Figure 2:**
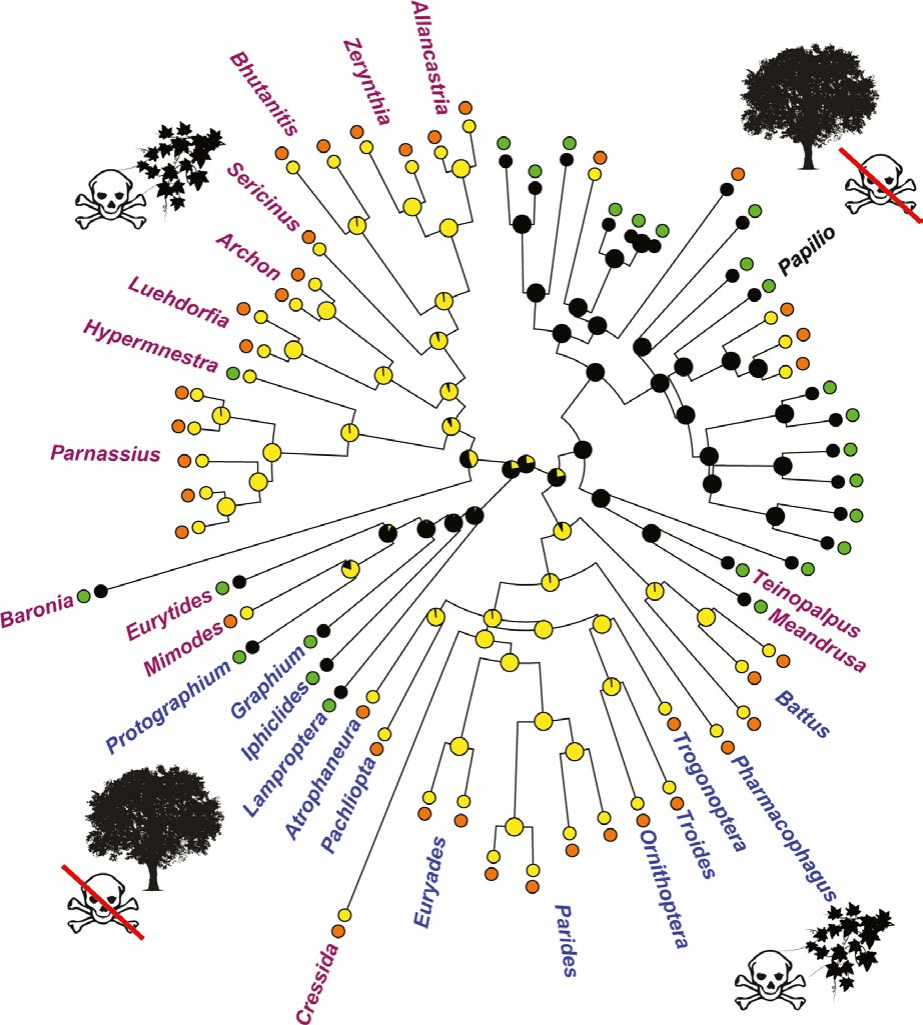
The combined effect of host plant toxicity and visual background strongly predicted the defense phenotypes of late‐instar caterpillars. Caterpillars on toxic plants with sparse vegetation (yellow, 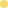) were aposematic, whereas those on nontoxic and densely vegetated plants (black, **●**) were cryptic. The phenotypes of late‐instar caterpillars were overlaid as aposematic (orange,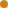 ) or cryptic (green, 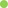). Lineages were either temperate (purple), tropical (blue), or globally distributed, that is, in both temperate and tropical areas (black,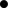)

The topology of our phylogenetic tree was congruent with the previously published global swallowtail butterfly phylogeny (Condamine et al., 2013). Ancestral trait mapping showed that the presence and nature of specific defensive color strategies were more or less uniform within monophyletic clades, indicating widespread phylogenetic conservation of defensive color strategies within swallowtail butterfly clades (Figures 1 and 2). However, the number and nature of defensive color strategies used by caterpillars were variable across lifestages, as expected: (a) Early‐ and late‐instar caterpillars feeding on toxic plants were aposematic, (b) caterpillars feeding on nontoxic plants predominantly masqueraded as bird droppings at early instars and were predominantly cryptic at late instars, and (c) more vulnerable larval stages exhibited all three types of color defense strategies (masquerade, crypsis, and aposematism), whereas the less vulnerable pupal stage exhibited only crypsis (Figure 1).

Masquerade was an ancestral defense for early instars of swallowtails, from which aposematism has evolved independently five times and crypsis two times (Figure 1a). Ancestral color defense of late instars was crypsis, from which aposematism was retained as a derived state in all clades that switched to feeding on toxic plants. In one lineage of *Papilio* (*Heraclides*), masquerade has been retained even in the last instar (Figure 1b). Most lineages masquerading at the early instars switched to crypsis and in two instances transitioned to aposematism in late instars (Figure 1). Crypsis was the ancestral defense strategy for the less vulnerable pupal stage, which has persisted in all swallowtail clades (Figure 1c).

Host plant toxicity and the visual background they offered to caterpillars were correlated with Papilionidae; that is, all toxic host plants were sparsely vegetated, whereas all nontoxic plants had dense vegetation (phylogenetic regression; *z* = 4.27, *p *<* *0.001). The correlated phytochemicals and visual background of caterpillars strongly predicted the color defense phenotype of late‐instar caterpillars: Aposematism was favored in the caterpillars feeding on sparsely vegetated toxic host plants, whereas crypsis was favored in caterpillars feeding on densely vegetated nontoxic plants (phyloglm: *z* = 4.52, *p *<* *0.001, Figure 2). However, geography did not predict the color defense phenotype and both tropical and temperate lineages exhibited both aposematic and cryptic strategies (phyloglm: *z* = 0.546, *p *=* *0.585).

## 4 DISCUSSION

The evolutionary history of Papilionidae offers itself as a grand global experiment, which revealed a hierarchy in color defenses. Crypsis appears to be the most effective strategy in the absence of motion and was phylogenetically conserved at the immobile pupal stage. In motile lifestages such as late‐instar caterpillars, the color defense phenotype was determined by the visual and phytochemical backgrounds wherein aposematism evolved under toxic and sparse vegetation and crypsis under nontoxic dense vegetation. However, early instars that have a small body size predominantly masqueraded as bird droppings at nontoxic dense backgrounds and shifted to crypsis with increase in body size at late instars. The repeated evolution of masquerade, crypsis, and aposematism in lifestages facing similar predation risk and comparable background conditions suggests that swallowtails took parallel evolutionary trajectories to reach similar outcomes and attain optimal defense phenotypes. Overall, color defenses in swallowtail butterflies have evolved as a result of a complex interaction between life history, phytochemical and visual backgrounds, and ancestry. Ontogenic color change is thus a life historical consequence of natural selection on complex life cycles, where changes in age‐dependent vulnerability to predation are coupled with change in protective coloration.

### 4.1 Evolutionary pattern of color defenses and Ontogenic Color Change in Papilionidae

The larval lifestages of Papilionidae switched their color defense strategies with ontogenic progress and a change in lifestage vulnerability (Figure 1). As expected, the early and late instars vulnerable to foliage‐gleaning insectivores exhibited multiple color defenses (aposematism, masquerade, and crypsis), whereas the less vulnerable immobile pupal stage employed a single tactic (crypsis; Figure 1c). The predominantly masquerading early instars transitioned to crypsis or aposematism with development and increase in body size. Increase in body size and movement gives away the cover of caterpillars masquerading as bird droppings. As the small early‐instar caterpillars grow to become large at late instars, the switch in the color defense was expected as high activity during foraging and movement before pupation along with a large body size attracts visually hunting predators, severely constraining protection by masquerade (Booth, 1990; Caro et al., 2016; Duarte et al., 2017; Endler, 1981; Grant, 2007; Skelhorn, Rowland, & Ruxton, 2010). Host plant toxicity and visual backgrounds significantly influenced the color defense strategy of vulnerable caterpillar stages, and lineages evolved a particular color defense depending on the background conditions (Figure 1). Caterpillars on toxic plants with sparse vegetation were aposematic, while those on nontoxic plants with dense vegetation either masqueraded as bird droppings or were cryptic (with two exceptions—*Hypermenstra helios* feeding on sparse toxic vegetation is cryptic, and *Papilio clytia* feeding on dense nontoxic vegetation is aposematic). These exceptions may arise due to local changes in predation risk and its relationship with other pressures such as higher susceptibility of aposematic species to parasitoids (Gentry & Dyer, 2002) and a trade‐off between the color defense and growth (Speed et al., 2010), which should be tested in future. Although the relative influences of host plant toxicity and visual backgrounds could not be teased apart because of their correlated nature, the importance of the visual backgrounds was evident at the pupal stage. All lineages irrespective of possessing toxins developed into cryptic pupae. Temperate lineages pupating on the ground were brownish to black in color, whereas tropical lineages pupating on vegetation were brownish to green in color (Supporting Information Figure S3). The nature of crypsis of pupae was dependent on local background conditions, and both temperate and tropical clades presumably blend their immobile pupae into the backgrounds to escape detection by predators and should be further investigated. Thus, phytochemical and visual backgrounds played a crucial role in determining the color defense in preadult stages of swallowtail butterflies.

While the color phenotypes of lineages at different lifestages were determined by the lifestage vulnerability and the visual and phytochemical backgrounds of lifestages, the broad evolutionary pattern may be influenced by several macroecological factors such as the latitudinal diversity gradient, environmental filters, and community dynamics that are known to influence the evolution of functional traits, such as color defenses (Elias & Joron, 2015; Joshi, Prakash, & Kunte, 2017; Kraft, Cornwell, Webb, & Ackerly, 2007; Losos, 2011). The widespread incidence of aposematism and crypsis across tropical and temperate lineages experiencing distinct environments indicates a limited role of phylogenetic constraint, geography, and macroevolutionary processes. It also indicates that although the composition of predator communities may be different, the comparable intensity of predation risk across tropical and temperate environments (Roslin et al., 2017) has led to similar evolutionary outcomes in the color defense strategies of swallowtail caterpillars (Figure 1).

### 4.2 Eco‐evo reciprocity and color defenses in Papilionidae

Adaptation to antagonists such as competitors, predators, parasitoids, and host plant defenses may be attained via similar or alternative strategies, and the coevolutionary response depends upon the magnitude of selection exerted by the antagonists relative to other selective pressures (Auld & Brand, 2017; Joshi & Thompson, 1995). Swallowtail caterpillars in a tritrophic interaction experience two crucial antagonists: (a) host plant defenses against herbivory and (b) threats from natural enemies such as predators and parasitoids (Greeney et al., 2012; Scriber et al., 1995; Takagi, Hirose, & Yamasaki, 1995). Evolution of color defenses in swallowtail butterflies elegantly illustrates the reciprocal interaction between ecology and evolution (Reznick & Ricklefs, 2009; Schoener, 2011). Lineages that underwent host shifts possibly to escape antiherbivory defenses of their host plants evolved color defenses according to the environment specified by the novel host: aposematism on sparse and toxic plants, and masquerade and crypsis on dense, nontoxic vegetation. The novel phytochemical and visual backgrounds after a host shift (evolutionary event) presumably brought a slew of change in natural enemies, resource utilization, and life history of swallowtail butterflies (Agosta, 2006). Lineages such as *Parnassius* and *Papilio*, which historically underwent host shifts (Condamine et al., 2012), exhibited both aposematic and cryptic phenotypes, whereas lineages coevolutionarily locked to a single host plant family displayed strong phenotypic conservatism with a single defense strategy (Figure 1c). The phylogenetic conservatism most probably arose due to a lack of ecological triggers such as altered phytochemical and visual backgrounds that lineages undergoing host shifts experienced. Thus, evolution of host plant shifts altered ecological settings which subsequently influenced evolution of color defenses satisfying the eco‐evo reciprocity loop.

## CONFLICT OF INTEREST

The authors have no conflict of interest to declare.

## AUTHOR CONTRIBUTIONS

NG and JJ conceived and designed the study and collected and analyzed the data. KK contributed to the experimental design and data analysis and wrote the manuscript with NG.

## DATA ACCESSIBILITY

The data associated with this publication are deposited at Dryad data repository and can be accessed at https://doi.org/10.5061/dryad.2q5b7gt.

## APPENDIX 1

### Character scoring of color defense phenotypes of larval life stages, host plant toxicity, geographic distribution of swallowtail butterflies

**Table:**

Sr. No.	Genus species	Lifestage phenotype			Factors			Reference to the life cycle
		Early^A^	Late^B^	Pupa^C^	Host toxicity^D^	Growth type^E^	Distribution^F^	
1	*Allancastria cerisyi*	3	3	2	1	1	1	http://www.leps.it/indexjs.htm?SpeciesPages/ZerynCeris.htm
2	*Allancastria deyrollei*	3	3	2	1	1	1	http://goran.waldeck.se/paindex3.htm
3	*Zerynthia polyxena*	3	3	2	1	1	1	http://www.butterfliesoffrance.com/html/Zerynthia%20polyxena.htm
4	*Zerynthia rumina*	3	3	2	1	1	1	https://en.wikipedia.org/wiki/Zerynthia_rumina
5	*Bhutanitis lidderaldi*	3	3	2	1	1	1	The Life histories of Asian butterflies Volume II
6	*Bhutanitis mansfieldi*	3	3	2	1	1	1	The Life histories of Asian butterflies Volume II
7	*Sericinus montela*	3	3	2	0	0	1	http://ftp.funet.fi/index/Tree_of_life/insecta/lepidoptera/ditrysia/papilionoidea/papilionidae/parnassiinae/sericinus/
8	*Archon apollinaris*	3	3	2	1	1	1	https://www.lepido-france.fr/parnassinae-du-kurdistan/
9	*Archon apollinus*	3	3	2	1	1	1	http://www.pyrgus.de/Archon_apollinus_en.html
10	*Luehdorfia japonica*	3	3	2	1	1	1	The Life histories of Asian butterflies Volume II
11	*Luehdorfia puziloi*	3	3	2	1	1	1	The Life histories of Asian butterflies Volume II
12	*Hypermnestra helios*	2	2	2	1	1	1	http://molbiol.ru/forums/lofiversion/index.php/t214757-600.html
13	*Parnassius apollo*	3	3	2	1	1	1	http://www.pyrgus.de/Parnassius_apollo_en.html
14	*Parnassius mnemosyne*	3	3	2	1	1	1	http://www.pyrgus.de/Parnassius_mnemosyne_en.html
15	*Parnassius eversmanni*	3	3	2	1	1	1	http://butterfliesofamerica.com/parnassius_eversmanni_thor_immatures.htm
16	*Parnassius phoebus*	3	3	2	1	1	1	http://www.pyrgus.de/Parnassius_phoebus_en.html
17	*Parnassius clodius*	3	3	2	1	1	1	https://www.butterfliesandmoths.org/species/Parnassius-clodius?page=2
18	*Baronia brevicornis*	2	2	2	0	0	0	Jones MT, Castellanos I, Weiss MR. Do leaf shelters always protect caterpillars from invertebrate predators?. Ecological Entomology. 2002 Dec 1;27(6):753‐7.
19	*Eurytides serville*	1	2	2	0	0	1	Tyler HA, Brown KS, Wilson KH. Swallowtail butterflies of the Americas. Scientific Publishers; 1994.
20	*Mimodes branchus*	3	3	2	1	1	0	http://caterpillars.myspecies.info/taxonomy/term/57299/media
21	*Protographium marcellus*	1	2	2	0	0	0	http://www.wikiwand.com/en/Protographium_marcellus
22	*Graphium agamemnon*	1	2	2	0	0	0	http://www.ifoundbutterflies.org/#!/sp/569/Graphium-agamemnon
23	*Iphiclides podalirius*	1	2	2	0	0	1	http://www.pyrgus.de/Iphiclides_podalirius_en.html
24	*Lamproptera meges*	1	2	2	0	0	0	http://www.pyrgus.de/Iphiclides_podalirius_en.html
25	*Atrophaneur aalcinous*	3	3	2	`	1	0	https://commons.wikimedia.org/wiki/Atrophaneura_alcinous#/media/File:Atrophaneura_alcinous_alcinous_larva.JPG
26	*Pachliopta neptunus*	3	3	2	0	0	0	http://ftp.funet.fi/index/Tree_of_life/insecta/lepidoptera/ditrysia/papilionoidea/papilionidae/papilioninae/atrophaneura/
27	*Cressida cressida*	3	3	2	1	1	0	http://lepidoptera.butterflyhouse.com.au/papi/cressid.html
28	*Euryades corethrus*	3	3	2	1	1	0	Tyler HA, Brown KS, Wilson KH. Swallowtail butterflies of the Americas. Scientific Publishers; 1994.
29	*Euryades duponchelli*	3	3	2	1	1	0	Tyler HA, Brown KS, Wilson KH. Swallowtail butterflies of the Americas. Scientific Publishers; 1994.
30	*Parides eurimedes*	3	3	2	1	1	0	http://images.peabody.yale.edu/lepsoc/jls/1970s/1977/1977-31(2)100-Young.pdf
31	*Parides neophilus*	3	3	2	1	1	0	http://images.peabody.yale.edu/lepsoc/jls/1970s/1977/1977-31(2)100-Young.pdf
32	*Parides anchises*	3	3	2	1	1	0	http://www.learnaboutbutterflies.com/Caterpillar%20-%20Parides%20anchises%20nephalion.htm
33	*Parides sesostris*	3	3	2	1	1	0	http://butterfliesofamerica.com/parides_sesostris_zestos_immatures1.htm
34	*Ornithoptera euphorion*	3	3	2	1	1	0	Sourakov AN. The Terrible Solomons. Tropical Lepidoptera Research. 2001;12:1‐4.
35	*Troides helena*	3	3	2	1	1	0	http://www.ifoundbutterflies.org/#!/sp/938/Troides-helena
36	*Trogonoptera brookiana*	3	3	2	1	1	0	Sourakov AN. The Terrible Solomons. Tropical Lepidoptera Research. 2001;12:1‐4.
37	*Pharmacophagus antenor*	3	3	2	1	1	0	http://images.peabody.yale.edu/lepsoc/jls/1990s/1996/1996-50(4)337-Parsons.pdf
38	*Battus belus*	3	3	2	1	1	0	http://ftp.funet.fi/index/Tree_of_life/insecta/lepidoptera/ditrysia/papilionoidea/papilionidae/papilioninae/battus/
39	*Battus philenor*	3	3	2	1	1	0	https://www.butterfliesandmoths.org/species/Battus-philenor
40	*Battus polydamas*	3	3	2	1	1	0	http://entnemdept.ufl.edu/creatures/bfly/polydamas.htm
41	*Meandrusa payeni*	1	2	2	0	0	0	On the life history of Meandrusa payeni (Boisduval) in Malaysia (Lepidoptera, Papilionidae) Tyo To Ga
42	*Meandrusa sciron*	1	2	2	0	0	0	On the life history of Meandrusa payeni (Boisduval) in Malaysia (Lepidoptera, Papilionidae) Tyo To Ga
43	*Teinopalpus imperialis*	1	2	2	0	0	1	The Life histories of Asian butterflies Volume II
44	*Papilio hospiton*	3	3	2	0	0	1	http://www.pyrgus.de/Papilio_hospiton_en.html
45	*Papilio protenor*	1	2	2	0	0	1	http://www.ifoundbutterflies.org/sp/733/Papilio-protenor
46	*Papilio polytes*	1	2	2	0	0	0	http://www.ifoundbutterflies.org/#!/sp/603/Papilio-polytes
47	*Papilio demodocus*	1	2	2	0	0	0	https://en.wikipedia.org/wiki/Papilio_demodocus
48	*Papilio demoleus*	1	2	2	0	0	0	http://www.ifoundbutterflies.org/sp/602/Papilio-demoleus
49	*Papilio bianor*	1	2	2	0	0	1	https://commons.wikimedia.org/wiki/File:Papilio_bianor_-_caterpillar_3_(HS).jpg
50	*Papilio maackii*	1	2	2	0	0	1	The Life histories of Asian butterflies Volume II
51	*Papilio machaon*	1	3	2	1	1	1	http://www.pyrgus.de/Papilio_machaon_en.html
52	*Papilio indra*	1	3	2	1	1	1	http://butterfliesofamerica.com/t/Papilio_indra_a.htm
53	*Papilio xuthus*	1	2	2	0	0	1	https://www.butterfliesandmoths.org/species/Papilio-xuthus
54	*Papilio anactus*	1	3	2	0	0	1	http://lepidoptera.butterflyhouse.com.au/papi/anactus.html
55	*Papilio dardanus*	1	2	2	0	0	0	http://www.mimeticbutterflies.org/gallery.php
56	*Papilio alexanor*	1	3	2	0	0	1	http://www.pyrgus.de/Papilio_alexanor_en.html
57	*Papilio glaucus*	1	2	2	0	0	1	https://www.butterfliesandmoths.org/species/Papilio-glaucus
58	*Papilio rutulus*	1	2	2	0	0	1	http://butterfliesofamerica.com/papilio_rutulus_immatures.htm
59	*Papilio multicaudatus*	1	2	2	0	0	1	http://butterfliesofamerica.com/t/Papilio_multicaudata_a.htm
60	*Papilio clytia*	1	3	2	1	0	0	http://www.ifoundbutterflies.org/#!/sp/601/Papilio-clytia
61	*Papilio anchisiades*	1	2	2	0	0	0	http://butterfliesofamerica.com/papilio_anchisiades_idaeus_immatures2.htm
62	*Papilio cresphontes*	1	1	2	0	0	1	https://www.butterfliesandmoths.org/species/Papilio-cresphontes
63	*Papilio thoas*	1	2	2	0	0	0	http://www.learnaboutbutterflies.com/Amazon%20-%20Heraclides%20thoas.htm

Character matrix legend	
Column	Character scoring
A	Early‐instar phenotypes are discretely scored as 1: masquerade, 2: cryptic, and 3: aposematic
B	Late‐instar phenotypes are discretely scored as 1: masquerade, 2: cryptic, and 3: aposematic
C	Pupal phenotypes are discretely scored as 1: masquerade, 2: cryptic, and 3: aposematic
D	The toxic nature of host plant family is binarily scored as 0: non‐toxic and 1: toxic
E	The growth type of the host plant family is binarily scored as 0: sparsely vegetated and 1: densely vegetated
F	The Papilionidae lineages are binarily scored according to their geographic range, 0: tropical and 1: temperate
