# Supplementary figures and images for: Evolution of ontogenic change in color defenses of swallowtail butterflies
Source: Ecol Evol. 2018 Sep 3;8(19):9751–63. doi: 10.1002/ece3.4426 (PMC6202720; doi:10.1002/ece3.4426)

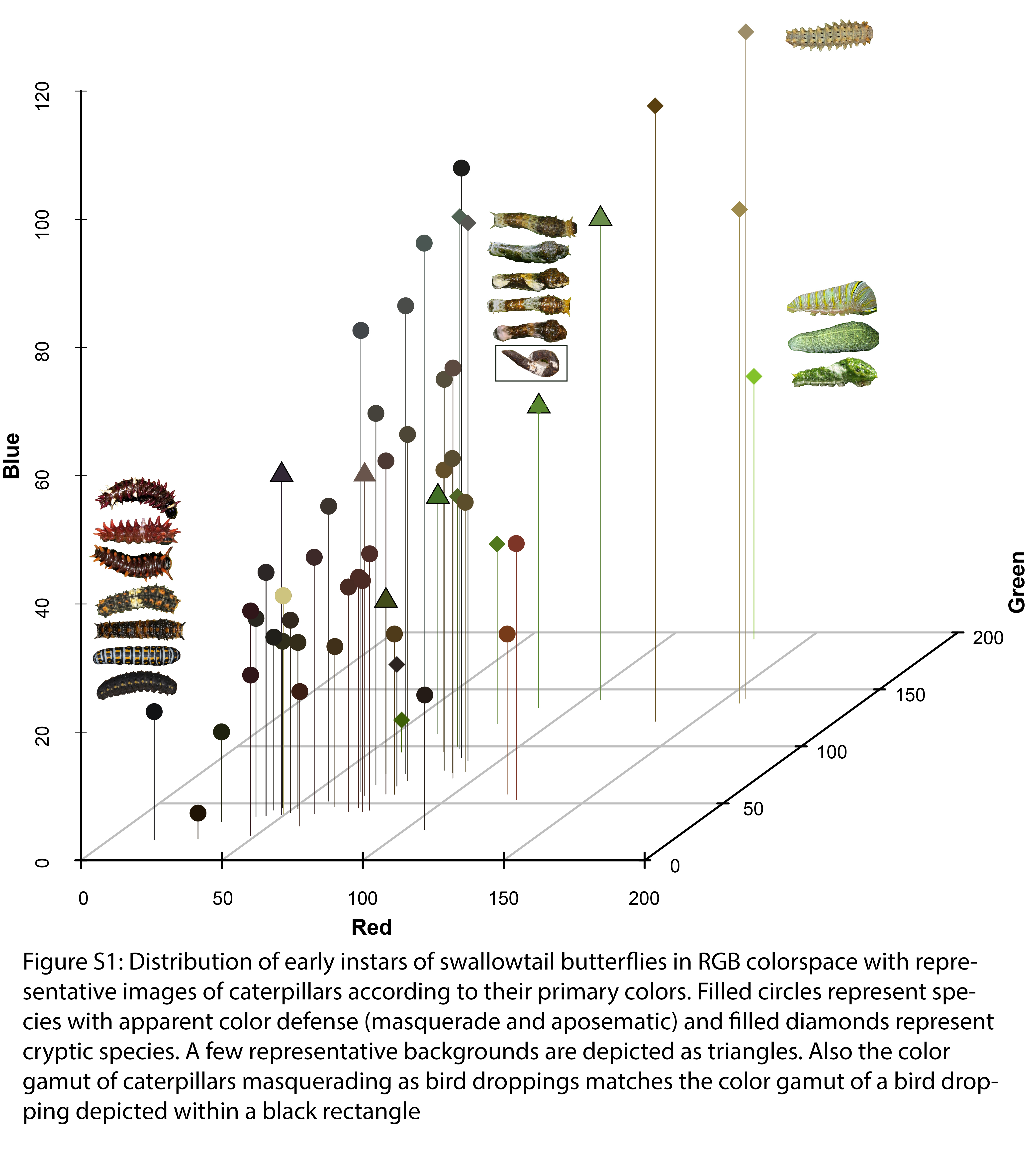

Supplement: Supplementary file 1 [file ECE3-8-9751-s001.tif]

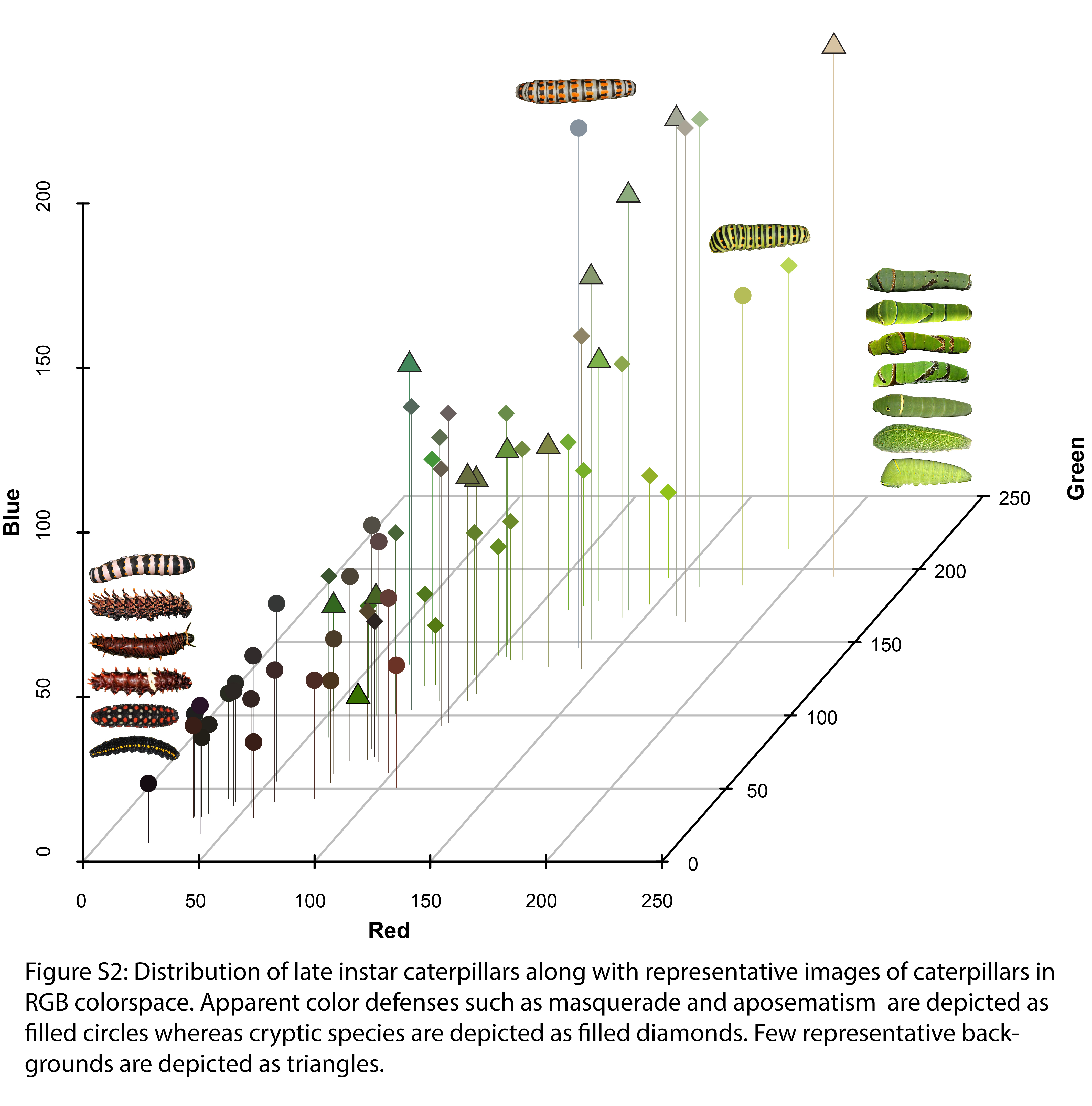

Supplement: Supplementary file 2 [file ECE3-8-9751-s002.tif]

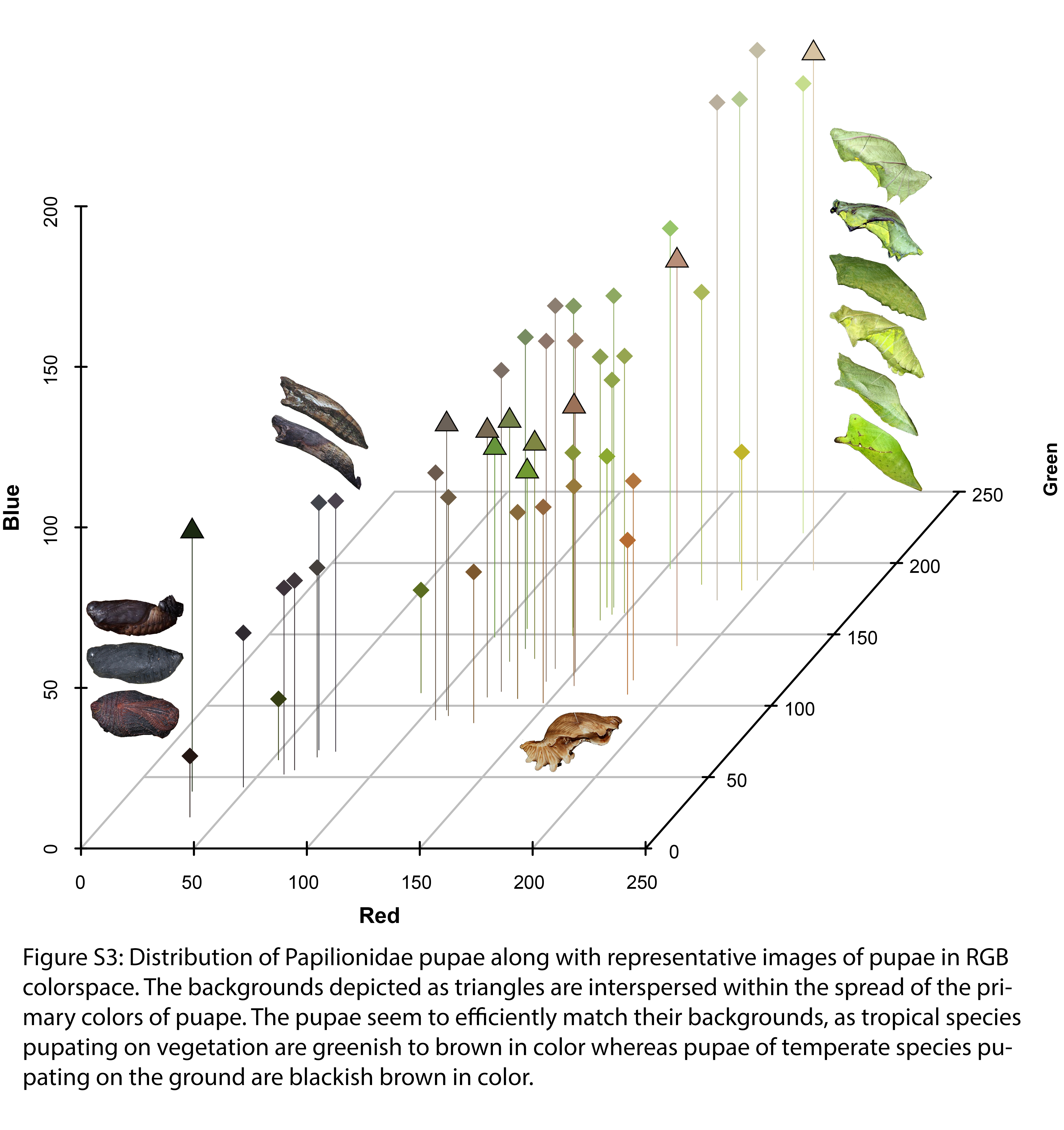

Supplement: Supplementary file 3 [file ECE3-8-9751-s003.tif]

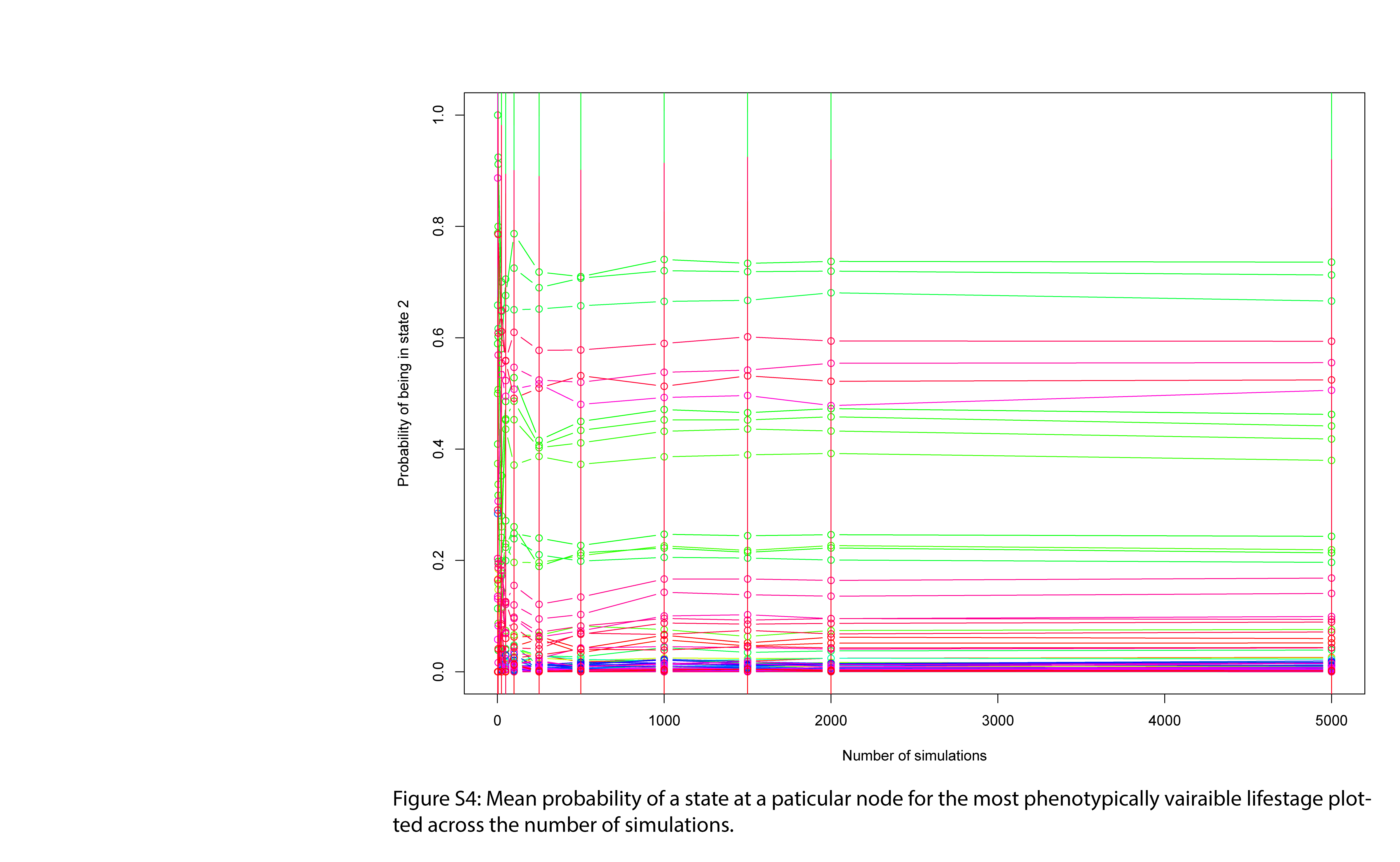

Supplement: Supplementary file 4 [file ECE3-8-9751-s004.tif]
